# Reduced and optimized trial designs for drugs described by a target mediated drug disposition model

**DOI:** 10.1007/s10928-018-9594-9

**Published:** 2018-06-08

**Authors:** A. Brekkan, S. Jönsson, M. O. Karlsson, A. C. Hooker

**Affiliations:** Department of Pharmaceutical Biosciences, Uppsala University, Box 591, 75124 Uppsala, Sweden

**Keywords:** Optimal design, Target mediated drug disposition, Monoclonal antibodies, Sampling time optimization, Model-based

## Abstract

Monoclonal antibodies against soluble targets are often rich and include the sampling of multiple analytes over a lengthy period of time. Predictive models built on data obtained in such studies can be useful in all drug development phases. If adequate model predictions can be maintained with a reduced design (e.g. fewer samples or shorter duration) the use of such designs may be advocated. The effect of reducing and optimizing a rich design based on a published study for Omalizumab (OMA) was evaluated as an example. OMA pharmacokinetics were characterized using a target-mediated drug disposition model considering the binding of OMA to free IgE and the subsequent formation of an OMA–IgE complex. The performance of the reduced and optimized designs was evaluated with respect to: efficiency, parameter uncertainty and predictions of free target. It was possible to reduce the number of samples in the study by 30% while still maintaining an efficiency of almost 90%. A reduction in sampling duration by two-thirds resulted in an efficiency of 75%. Omission of any analyte measurement or a reduction of the number of dose levels was detrimental to the efficiency of the designs (efficiency ≤ 51%). However, other metrics were, in some cases, relatively unaffected, showing that multiple metrics may be needed to obtain balanced assessments of design performance.

**Electronic supplementary material:**

The online version of this article (10.1007/s10928-018-9594-9) contains supplementary material, which is available to authorized users.

## Introduction

Monoclonal antibodies (mAbs) are increasingly present in pharmaceutical development pipelines [1, 2]. As a result, methods to characterize mAb disposition have received a great deal of attention and pharmacometric models describing the pharmacokinetics (PK) and pharmacodynamics (PD) of mAbs are frequently published [3–5]. Many mAbs display target-mediated drug disposition (TMDD), arising when binding to a target affects the disposition of the mAb. Such interactions may result in nonlinear PK at low drug concentrations and linear PK at higher concentrations due to saturation of target-mediated clearance pathways [6, 7]. Additionally, for mAbs against soluble targets, elimination may depend on the nature of the formed antibody–target complex, which may be cleared differently than the unbound mAb [8].

Pharmacometric models have been used to describe distribution, binding and elimination of mAbs and the general TMDD model offers a semi-mechanistic interpretation of the disposition of mAbs [9–11]. This model describes the formation of a drug–target complex and is often unidentifiable since samples may not be available for all analytes described by the system or at time-points required for accurate parameter estimation. Additionally, rate parameters in the model can vary greatly in magnitude, where drug elimination can take weeks while drug–target binding may occur within minutes, causing model instability. To ensure identifiability and avoid model instability, approximations of the general TMDD model have been suggested, e.g. the quasi-equilibrium (QE) approximation, assuming rapid equilibrium between drug, receptor and drug–receptor complex making the TMDD model’s reliance on rich information in the transition phase less critical for identifiable parameter estimates [12, 13]. These assumptions can be valid when association and dissociation of the drug and target are rapid compared to other PK processes. When the internalization and degradation of the drug–target complex is equal to the elimination of the target or when no low dose information is available, the TMDD model approximations can be further simplified to a Michaelis–Menten (MM) approximation for easier estimation of model parameters [14].

Optimal design (OD) methodology for nonlinear mixed effects models has been developed to maximize the information gathering potential of experiments in drug development, to reduce the number of samples required for accurate parameter estimation and to generally optimize designs with respect to sampling time points, doses to be administered and other design variables [15–17]. Model-based OD is not limited to the optimization of clinical trial designs of different phases. OD can also be used in most development phases for mAbs and other biologics, including preclinical development, starting with the optimization of the drug properties, i.e. identifying the optimal binding affinity to the pharmacological target to identifying the optimal administration route/site to optimization of the trial properties as demonstrated herein [18]. Further, OD is a flexible methodology that can be performed on an individual (fixed effect) or population (mixed effect) level where the latter considers parameters for the characterization of variability, such as inter-individual variability. Population OD is perhaps more relevant in a clinical setting, where several patients in several groups are given an intervention [17]. Using OD, it may be possible to reduce rich study designs for mAbs without significantly reducing the amount of information collected or negatively affecting model performance [17, 19, 20]. Further, if the same decisions regarding drug development can be made with less extensive studies, then the use of such studies can easily be advocated on ethical and economic grounds.

Study designs for mAbs against soluble targets are often rich and include the sampling of multiple analytes at a number of different dose levels and for a lengthy period of time. This work was performed to determine whether a reduction (fewer samples, shorter duration, fewer dosing levels and fewer measured analytes) and optimization of these rich designs used to study biological drugs characterized by a TMDD model could give adequate information for drug understanding and development decision making.

To investigate these questions, we utilize a published rich study design and TMDD model describing the QE approximation applied for omalizumab (OMA), an anti-immunoglobulin E (IgE) antibody [21, 22]. The initial rich design and model were used to determine the consequences of reducing and optimizing the study design on design efficiency, parameter precision, precision of free target level predictions at certain time-points and on a hypothetical go/no-go decision regarding dose selection.

## Methods

This work serves as a general illustration of the potential of reducing and optimizing study designs for data characterized by a TMDD model in any stage of drug development. Further, we illustrate the use of multiple metrics for design evaluation. Thus, the goal of this work is not focused particularly on OMA. The model, parameter estimates and a reference study design previously published used in this work function as examples [21]. This reference study design and several alternative designs were evaluated and optimized.

### Population model

The model used in this work is a simplified version of the QE approximation describing the binding of OMA to IgE and the formation of an OMA–IgE complex. The model was simplified by removing covariate relationships (body weight and baseline IgE levels) and correlation between parameters. A detailed model description and parameter values are provided as supplementary information (Supplementary material Appendix 1).

### Reference and reduced study designs

**Fig. 1:**
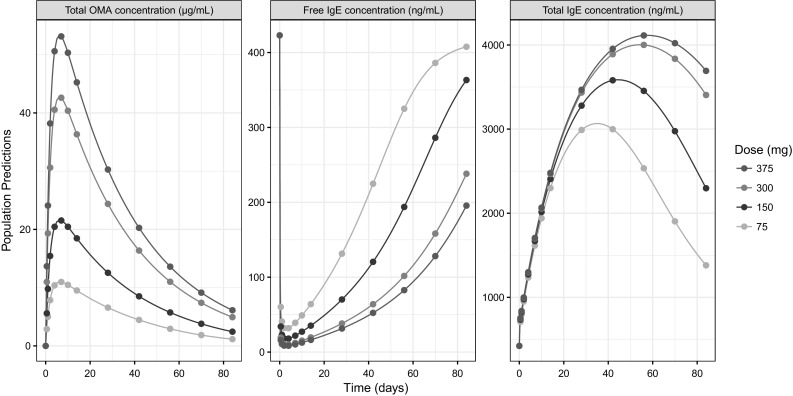
Population predictions of C_OMA,T_ (left panel), C_IGE,F_ (middle panel) and C_IGE,T_ (right panel) versus time at four different dose levels (75, 150, 300, 375 mg). The points indicate the reference study design sampling times

The reference study design in this work (illustrated in Fig. 1) was a single-dose clinical study in which 48 individuals were allocated to one of four dose groups (75, 150, 300 and 375 mg) receiving a subcutaneous (SC) dose of OMA. Thirteen blood samples were collected at 0, 0.5, 1, 2, 4, 7, 10, 14, 28, 42, 56, 70 and 84 days after administration and each sample was analysed for C_OMA,T_, C_IGE,F_ and C_IGE,T_ resulting in a total of 39 observations per individual. The data collected in accordance with the reference study design was used for model building and validation in the work by Hayashi et al. [21].

The reference study design was reduced and optimized to answer the following questions;

1. What is the effect of sampling duration given the time scale of OMA half-life (18.2 days) considering a maximum sampling times of 2, 14, 28 or 84 days?
2. What is the effect of reducing the total number of sampling times?
3. What is the effect of reducing the number of dose groups in addition to the number of samples?
4. What is the effect of not sampling one of the analytes?

### Design optimization

The R package PopED (version 0.2.0) was used to perform the optimizations of the evaluated study designs (Table 1) [23]. Optimization speed was increased (~ 2 fold) by writing the model functions in the C language and compiling into a dynamically linked library file [24].

**Table 1:** The initial sampling schedule for the reference study from Hayashi et al. [21] and for evaluated reduced designs

Design	Description	Total number of samples	Sampling times (days)	Observations/individual	Dose levels^a^
Reference design					
1	Reference design	1872	0, 0.5, 1, 2, 4, 7, 10, 14, 28, 42, 56, 70, 84	39	4
Reducing sampling duration					
2	Sampling for 2 days	1296	0, 0.25, 0.5, 0.75, 1, 1.25, 1.5, 1.75, 2	27	4
3	Sampling for 14 days	1296	0, 0.5, 1, 2, 4, 6, 8, 10, 14	27	4
4	Sampling for 28 days	1296	0, 0.5, 1, 2, 4, 7, 10, 14, 28	27	4
Removal of dose groups					
5	Removing 2 dose groups^b^	936	0, 0.5, 1, 2, 4, 7, 10, 14, 28, 42, 56, 70, 84	39	2
Removal of samples					
6	Minus 4 samples per analyte	1296	0, 0.5, 1, 2, 4, 7, 10, 14, 28^c^	27	4
7	Minus 6 samples per analyte	1008	0, 0.5, 1, 4, 7, 28, 84	21	4
8	Minus 8 samples per analyte	720	0, 0.5, 7, 14, 84	15	4
Removal of an analyte					
9	No total IgE sampling	1248	0, 0.5, 1, 2, 4, 7, 10, 14, 28, 42, 56, 70, 84	26	4
10	No free IgE sampling	1248	0, 0.5, 1, 2, 4, 7, 10, 14, 28, 42, 56, 70, 84	26	4
11	No total omalizumab sampling	1248	0, 0.5, 1, 2, 4, 7, 10, 14, 28, 42, 56, 70, 84	26	4
12	No total IgE minus 8 samples per remaining analyte	480	0, 0.5, 1, 4, 84	10	4

The designs are grouped by the aspect explored for influence on performance: sampling duration, number of dose groups, number of samples and analyte omission

^a^The dose levels were 75, 150, 300 and 375 mg

^b^The dose levels were 75 and 150 mg

^c^During optimization, the final sampling time was permitted to exceed 28 days, in contrast to design 3 where the final sampling time was fixed at 28 days

When optimizing designs, PopED maximizes the Fisher Information Matrix (FIM) [15]. According to the Cramer-Rao inequality, maximizing the FIM with respect to design variables results in a lower bound of the estimate of the variance–covariance matrix for model parameter estimates:

$$\varvec{COV}\left( {\varvec{\varTheta},\varvec{X}} \right) \ge \varvec{FIM}(\varvec{\varTheta},\varvec{X})^{ - 1}$$

where **Θ** are population parameters in the model and **X** are design variables. Maximizing the determinant of the FIM, known as the D-optimal criterion, minimizes the expected variance–covariance matrix of model parameters **Θ** fit to data from design **X**. The reduced FIM was used assuming no correlation between the covariance matrix of fixed and random effects parameters [23]. Each design was optimized using a D-optimal criterion with respect to sampling times. Sample times were allowed to vary between day 0 and the end of the experiment. A Line Search (LS) optimization algorithm was used, which discretized the allowed sampling region. A grid size of 84 in the sampling time optimizations was used ensuring that a sampling time could be selected every day for the entire time span of the reference study design. Clustering of sample times was allowed during the optimizations [25]. The optimized designs were compared to their non-optimized counterparts to evaluate the effects of optimization.

### Evaluation of designs

#### Efficiency

D-efficiency was used to compare investigated designs to the original trial design, defined as [26]:

$${\text{D-efficiency}} = \left( {\frac{{\left| {{\mathbf{FIM}}} \right|}}{{\left| {{\mathbf{FIM}}^{*} } \right|}}} \right)^{1/p}$$

where p is the number of estimated parameters in the model and * denotes the reference design. Efficiency is a metric of the amount of information expected in a trial design when compared with a reference design. For example, a competing design with an efficiency of 0.5 indicates that a trial would need to be replicated twice (or the number of subjects doubled) in order to achieve parameter estimates with equal precision as those obtained in a trial with a reference design. Additionally, efficiency can be interpreted as the number of individuals needed in an optimized or evaluated design to match a reference design.

#### Parameter precision

The stochastic simulation and estimation (SSE) functionality in Perl-speaks-NONMEM (PsN) was used to determine the parameter precision resulting from the study designs [27]. One hundred datasets were simulated and estimated from the reference model but using the different trial designs, resulting in 100 parameter vectors from each design. These vectors were used to calculate the parameter uncertainty for each design as relative standard error (%RSE). Overall parameter precision was defined as the average %RSE of all the parameters in the model (fixed and random effect parameters) for each competing design, which was compared to the same value calculated for the reference study design. All simulations and estimations were run in NONMEM version 7.3 [28]. Similar results could, most likely, also be obtained via the FIM without simulation [29]. In this work we investigate parameter uncertainty via SSE to investigate realized uncertainty (instead of asymptotic uncertainty) and to avoid assumptions about non-biased parameter estimates. This should further differentiate this design evolution method from the efficiency metric discussed above.

#### Population prediction areas

To investigate the effect of parameter uncertainty on predictions of the typical individual from the model, ninety-five percent population prediction areas (PPA) were generated for C_IGE,F_ population predictions versus time for each design investigated. For each parameter vector created in the SSE described above, C_IGE,F_ population predictions for the time points of the initial full sampling schedule were simulated given a dose of 150 mg. For each time-point the upper 97.5th and lower 2.5th percentiles were obtained resulting in a 95% prediction interval. The sum of the resulting 95% prediction intervals were then used to calculated the PPAs for the time span 0–84 days. Additionally, a PPA ratio (PPAR) was computed by dividing the PPA of the reference study design by the PPA of the competing designs. Ratios below 1 indicate PPAs that are wider with competing designs. In addition, specific attention was paid to the prediction at 14 days post-dose, considering that this is the time of subsequent OMA administration when OMA is dosed every 2nd week. The treatment goal of OMA is to maintain C_IGE,F_ beneath a certain threshold concentration, therefore, it may be important to maintain a tight prediction interval corresponding to the time-point of interest (in this case 14 days).

#### Go/no-go decision

**Fig. 2:**
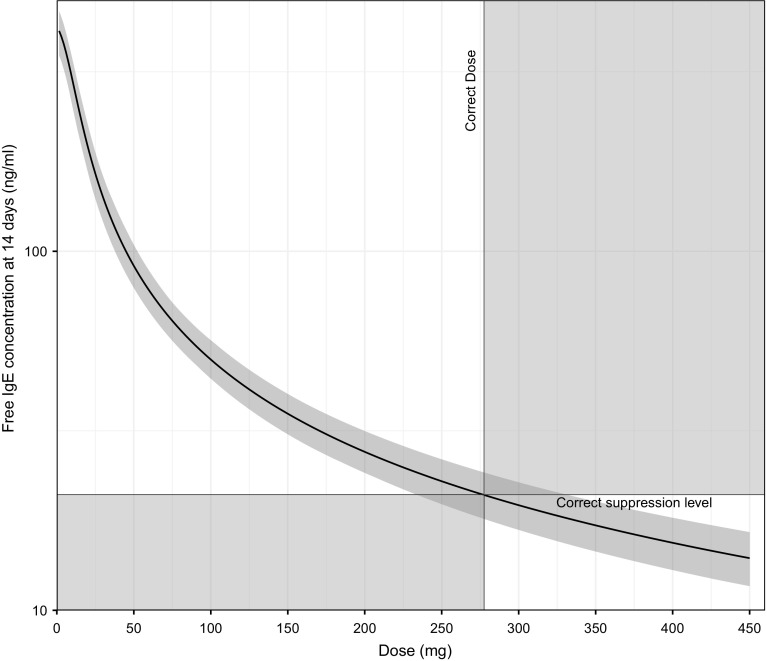
Population C_IGE,F_ predictions at 14 days versus dose. This is a schematic depiction of how incorrect decisions were defined. The black curve is population C_IGE,F_ based on the true parameter estimates. The grey shaded region around the line depicts the 95% C_IGE,F_ confidence interval resulting from the same population C_IGE,F_ predictions based on estimated parameter vectors (in this case derived from an SSE). The black vertical line represents the true dose (277.5 mg) resulting in a 95% C_IGE,F_ reduction. A dose lower than the true dose yielding a C_IGE,F_ reduction below 95% or a dose higher than the true dose yielding a C_IGE,F_ reduction above 95% was defined as an incorrect decision (shown by the red shaded areas)

A, potentially, more understandable design performance metric was derived as the probability of making an incorrect decision with regards to dose-selection utilizing the trial designs and reference model. First, the dose (true dose) resulting in a 95% reduction of C_IGE,F_ from the baseline concentration at 14 days (corresponds to a reduction from 422.82 ng/mL to the clinically relevant concentration of 21 ng/mL) was calculated using the reference parameter estimates (true parameters) [21]. Secondly, using the SSE parameter vectors, derived above, the population prediction of C_IGE,F_ at 14 days was computed for 300 doses ranging from 1.5 to 450 mg. If a prediction for a dose higher than the true dose resulted in C_IGE,F_ > 21 ng/mL it was defined as an incorrect decision; the same was true when a prediction for a dose lower than the true dose resulted in C_IGE,F_ < 21 ng/mL (Fig. 2). Finally, for each design, the number of incorrect decisions was divided by the total number of C_IGE,F_ predictions resulting in the probability of an incorrect go/no-go decision.

## Results

Table 2 presents the efficiency, parameter precision and PPARs for the competing designs when evaluated and when optimized. Optimizations took between 53 min and 14 h with the selected settings.

**Table 2:** Average estimated relative standard error (%RSE), efficiency and population prediction area ratios (PPAR) for each of the evaluated designs grouped by the aspects explored for influence on performance: sampling duration, number of dose groups, number of samples and analyte omission

Design	Description	Total number of samples	Sampling times (days)	Average estimated %RSE	Efficiency (%)	PPAR
Reference						
1	Reference	1872	0 0.5 1 2 4 7 10 14 28 42 56 70 84	14	100	1.00
1O	1 Optimized	1872	0 0 0.5 1 11 11 11 11 11 11 46 71 84	13	104	1.11
Reducing sampling duration						
2	Sampling for 48 h	1296	0 0.25 0.5 0.75 1 1.25 1.5 1.75 2	>1900	7	0.08
2O	2 Optimized	1296	0 0.14 0.14 0.81 0.81 2 2 2 2	>1600	11	0.08
3	Sampling for 14 days	1296	0 0.5 1 2 4 6 8 10 14	27	46	0.39
3O	3 Optimized		0 0.17 4.89 5.06 5.23 14 14 14 14	22	53	0.61
4	Sampling for 28 days	1296	0 0.5 1 2 4 7 10 14 28	18	67	0.74
4O	4 Optimized	1296	0 0.33 7.67 7.67 7.67 7.67 28 28 28	17	75	0.80
Removal of dose groups						
5	2 dose groups removed	936	0 0.5 1 2 4 7 10 14 28 42 56 70 84	20	49	0.61
5O	5 Optimized	936	0 0 0.5 1 10 10 10 10 10 40 40 72 84	20	51	0.62
Removal of samples						
6	Minus 4 samples	1296	0 0.5 1 2 4 7 10 14 28	18	67	0.74
6O	6 Optimized	1296	0 24 24 11 11 11 11 47 83	16	87	0.85
7	Minus 6 samples	1008	0 0.5 1 4 7 28 84	16	73	0.93
7O	7 Optimized	1008	0 1 11 11 11 46 83	16	76	0.96
8	Minus 8 samples	720	0 0.5 7 28 84	18	62	0.89
8O	8 Optimized	720	0 0.5 12 12 72	18	63	0.94
Removal of an analyte						
9	No total IgE	1248	0 0.5 1 2 4 7 10 14 28 42 56 70 84	18	42	0.90
9O	9 Optimized	1248	0 0 0.5 1 1 11 11 11 11 36 40 74 84	18	45	0.86
10	No free IgE	1248	0 0.5 1 2 4 7 10 14 28 42 56 70 84	22	36	0.36
10O	10 Optimized	1248	0 0 0.5 1 11 11 11 11 46 46 73 84 84	21	38	0.40
11	No total omalizumab	1248	0 0.5 1 2 4 7 10 14 28 42 56 70 84	23	33	0.83
11O	11 Optimized	1248	0 0 0.5 1 1 12 13 13 13 56 72 72 84	24	34	0.92
12	No total IgE minus 8 samples per remaining analyte	480	0 0.5 1 4 84	43	31	0.73
12O	12 Optimized	480	0 0.5 10 39 83	23	38	0.83

### Efficiency

All reported efficiencies are a result of the comparison with the reference study design. Keeping the reference study design duration but reducing the number of samples from 1872 (design 1) to 1296 (designs 6 and 6O) and to 1008 (designs 7 and 7O) resulted in efficiencies of 87 and 76%, respectively, for optimized designs. Reducing the number of dose groups from four to two (including the 75 mg and 150 mg dose groups) resulted in an efficiency of 51% when optimized (design 5O).

Efficiency of the designs increased with increasing duration of sampling (while the number of samples was kept at 1296). The efficiency of designs 2 and 2O (optimized) were 7 and 11%, respectively, where sampling was only allowed for up to 2 days. When the final sampling time was extended to 14 days the efficiency was 46% (design 3) and 53% when optimized (design 3O). Further increasing the maximum sampling time to 28 days improved the efficiency further (67% for design 4 and 75% for the same design when optimized, design 4O). Efficiency improved to 87% when the maximum sampling duration was extended to 84 days and the design was optimized (design 6O). Before optimization designs 4 and 6 were identical, but differences were seen during optimization as the maximum sampling time was allowed to be larger than 28 days in design 6.

Designs 9O, 10O and 11O omitted C_IGE,T_, C_IGE,F_ or C_OMA,T_ measurements entirely, respectively. None of the designs resulted in efficiency that was > 45% and slightly improved by optimization. Reducing the number of samples per analyte from 13 to 5 and omitting C_IGE,T_ measurements entirely resulted in an efficiency of 31% (design 12).

#### Parameter precision

In line with the efficiency results, the average estimated %RSE increased with decreasing sampling duration. When sampling was allowed over 2 days (designs 2 and 2O) the design resulted in an average parameter %RSE in excess of 1000%. This average value was influenced by the %RSE of OMA clearance (CL_OMA_) and complex clearance (CL_COMP_) and their associated inter-individual variability (IIV) parameters; calculating the average %RSE without these parameters resulted in an average %RSE < 30%.

Having up to 8 fewer samples per individual (designs 8 and 8O) than the reference study design had a small impact on the average %RSE (increased by < 4% compared with the reference study design). Parameter imprecision increased by approximately 6 percentage points when two dose groups were omitted.

Average %RSE was < 25% when omitting measurements of C_IGE,T_, C_IGE,F_ or C_OMA,T_. However, the %RSE for the fixed effect parameter *α* was > 100% in designs 10 and 10O, where C_IGE,F_ was omitted. The other designs omitting analytes entirely did not have any fixed effect parameter %RSE > 30% when optimized. Design 12 featuring 5 measurements of C_OMA,T_ and C_IGE,F_, respectively, resulted in an estimated average %RSE of 43.3% and decreased to approximately 23% by optimization.

### Population prediction areas

**Fig. 3:**
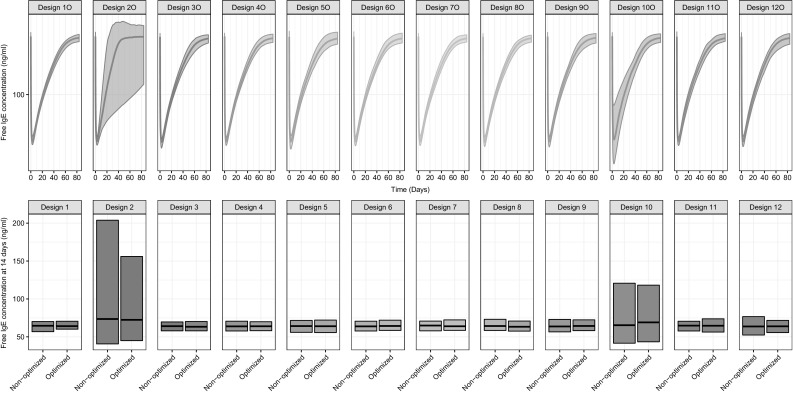
Free IgE concentration population predictions versus time for each of the optimized designs (upper panels) and at 14 days post-dose for optimized and non-optimized designs (lower panels), following a 150 mg dose. In the top panel the central line is the median prediction, and the shaded area illustrates the 95% population prediction areas (PPA). In the bottom panel the black horizontal line is the median prediction and the boxes represent the 95% prediction interval

The majority of the evaluated designs had a PPAR between 0.8 and 1 indicating a prediction area up to 25% larger than the reference study design (Table 2, Fig. 3). Designs 2 (sampling for 2 days), 3 (sampling for 14 days) and 10 (no C_IGE,F_ measured), resulted in a PPA that was considerably larger than the reference study design (PPAR of 0.08, 0.61 and 0.40, respectively for optimized designs). PPARs improved drastically with optimization of Design 3. Design 10 had an unexpectedly large PPA, likely traced back to the parameter uncertainty of α, described above. At 14 days, the population predictions of C_IGE,F_ resulting from all designs excluding designs 2 and ten were relatively tight (Fig. 3).

### Go/no-go decision

**Fig. 4:**
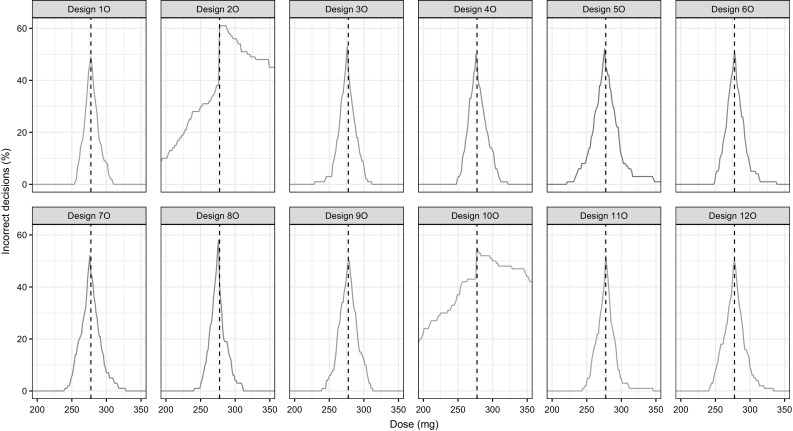
The probability of making an incorrect decision defined as incorrectly identifying doses which should result in a C_IGE,F_ reduction of 95%. The dashed vertical black line indicates the true dose of 277.5 mg

The true dose resulting in a 95% reduction of population predictions of C_IGE,F_ from baseline at 14 days was 277.5 mg. All optimized designs apart from designs 2O and 10O resulted in an erroneous decision in less than 5% of the simulations. Designs 2O and 10O resulted in 21.2 and 23.0% erroneous decisions, respectively. With increasing dose the probability of making an incorrect decision increased for all designs until it reached a maximum around the true dose and then decreased again (Fig. 4). Given a symmetric parameter uncertainty distribution and an unbiased design, a 50% probability of making a correct decision at the true dose is the best that can be achieved due to the way incorrect decisions are defined (C_IGE,F_ predictions above the reduction threshold at the true dose is considered to be incorrect but C_IGE,F_ predictions below the threshold at the true dose are not considered incorrect).

## Discussion

Sampling schedules for mAb studies are often long due to the typically long half-life of mAbs [30]. Further, both the amount of sampling and doses studied are rich in order to capture the complex kinetics of the system. Thus, a reduction in the duration, the number of samples and dose levels investigated in trials featuring mAbs may be beneficial from a cost and patient burden perspective, provided that the impact is minimal, and accurate decisions regarding drug development can be made. The work performed herein demonstrates the potential application of OD to evaluate sampling design reductions for systems described by TMDD models. Further, this work demonstrates the importance of utilizing a number of different metrics to evaluate the information loss when reducing trials.

For the example system investigated, it was possible to reduce the number of samples in the study by 30% while still maintaining an efficiency of 87%. Reducing the number of samples by 62% resulted in lower efficiency (63%), indicating the need to increase the number of individuals in the study with this reduced design to match the original rich design but this reduction had little or no effect on the other design metrics investigated including the probability to make an incorrect go/no-go decision.

With the same number of total samples, a reduction in the sampling duration was detrimental to the efficiency of the designs. Although the efficiency was impacted negatively, a reduction of the sampling duration from 84 to 14 days resulted in acceptable performance with regards to the other performance metrics, at least when optimized. Sampling for 14 days without optimization resulted in relatively poor PPAR and this design benefited most with optimization. A 2 day sampling design was found to be very poor, especially with regards to estimates of CL_OMA_ and IgE clearance (CL_IGE_) and their corresponding IIV. These results were not unsurprising, given the half-life of mAbs. Since therapeutic mAbs have the same general structure (immunoglobulin G) the expectation is for them to undergo the same intrinsic elimination and as such a 2 day sampling schedule would probably have been avoided from the outset. However, for molecules for which little is known, a priori, a very short sampling duration may be evaluated.

Omission of measuring any analyte was detrimental to the efficiency of the designs. However, omitting sampling of C_IGE,T_ (designs 8 and 8O) did not have a substantial impact on the other evaluated metrics, indicating local identifiably with no C_IGE,T_ samples (an identifiability analysis as described by Gibiansky et al. could also be applied here [13]).

The inclusion of multiple metrics to gauge the performance of alternative designs is important to get a balanced assessment of their performance. Metrics such as efficiency may not be easily communicated to stakeholders in drug development and alternative metrics such as the probability of making an erroneous decision may be easier to use in conveying the intended message. Even though the decision metric in this work is simple, it serves as an example of how parameter precision propagates to other aspects of trial performance. All metrics identified the best design as being the optimized reference (design 1O) and design 2 as the worst design. However, in some cases, there was a deviation between design performances evaluated with different metrics. Deviance of the PPAR and go/no-go metric results from the efficiency and parameter uncertainty results are not entirely unexpected since the PPAR and go/no-go metrics depend solely on the fixed-effect parameters while the other metrics consider random-effects parameters. Deviation between the efficiency metric and average %RSE can also be expected as efficiency takes into account the covariance between uncertainty in parameter estimation, something ignored by average %RSE. Optimizing designs based solely on increasing efficiency may make viable trial designs unattractive but by looking at several alternative metrics acceptable designs may be identified that do not meet the criteria for efficiency. The optimization of designs with OD is flexible and almost any conceivable metric could be optimized. For instance, to obtain the most accurate prediction of IgE at a certain time-point an optimization using a C-optimality considering the most informative parameters for IgE at that time-point could have been used. Alternative optimizations using different criterions were omitted due to the additional complexity that they entail versus “standard” D- or Ds-optimal design. However, in this work we focused on optimizing a global metric, parameter precision, in order to be able to draw conclusions about multiple performance metrics related to parameter precision.

Many mAbs may exhibit nonlinear PK profiles and thus optimal sampling times will vary with dose. However, antibodies against soluble targets, as investigated here, can display nonlinear PK but do not tend to do so [31]. While the model for OMA used in this work is a TMDD model capable of describing nonlinearity, the measured OMA analyte was the total concentration which appeared to be linear. If free OMA were measured, nonlinear PK may have been observed at certain doses and optimizing dose may be more fruitful than optimizing the sampling times. In this example, optimizing doses had a marginal effect on the efficiency of the reference study design (results not shown). The results in this work are, however, comparable to results published by Davda et al. where a sparse sampling schedule contained similar information as a rich sampling schedule for mAbs described by a 2-compartment linear elimination model [32]. Additionally, when fewer moieties are sampled, model identifiability issues may be circumvented by using further simplifications of the model such as a Michaelis–Menten (MM) approximation. Additional work is needed to determine whether it is possible to translate the results of this work to models in the same hierarchy such as the MM approximation or the more complex full TMDD model.

The design used as a reference design here is not one that can be recommended for late phase analyses but it is typical of early phase data for monoclonal antibodies, although such studies may often feature dose escalation [33–35]. Ideally, multiple models and trial designs would have been explored to obtain a more generalizable conclusion. This was not feasible given the lengthy optimization times. However, many mAbs have similar disposition and the results obtained by this work may be applicable to other mAbs against soluble targets, but warrants further study.

The model employed in this work was a reduction of the original model where covariate relationships and correlations between random effects were excluded. These simplifications were deemed acceptable since model performance was not evaluated and the specific aim of this work was to illustrate a methodology rather than performing a reduction and optimization of trial design specifically for OMA. Performing OD with models including covariate relationships comprises integration over all potential covariate values and substantially increases the optimization times. A more feasible workflow for the optimization would be to identify an interesting reduced design among competing designs to be further optimized assuming distributions of covariates.

In this work clustering of sample times, rarely feasible in practice, was allowed in the design optimizations. Clustering occurs when a design attempts to minimize the signal-to-noise ratio but usually results in a less informative design as error generating mechanisms are not considered. Clustering can be avoided in the optimization at the outset by incorporating autocorrelation into the model or through the use of sampling windows [25, 36]. However, incorporation of an autocorrelation model frequently increases optimization times and was therefore omitted in this work. The results of the optimizations can therefore be regarded as the best possible designs for the model, parameter values and scenarios investigated. Specific design scenarios that perform well in these examinations would then be further improved upon, through more elaborate and time-consuming optimization methods.

When optimizing a study design during the development of a novel compound the parameter estimates are not known a priori. Thus, reducing the number of samples and optimizing those samples, based on specific parameter values, may have unintended consequences if those parameter values are misspecified. Different global optimization criteria, such as the ED-optimal criteria where uncertainty in parameter values are accounted for should increase robustness in the optimization and can be advocated for scenarios where knowledge of the compound of interest is limited [37]. Further, the model structure to describe the disposition of compounds of a similar nature, such as mAbs, may be comparable and prior information gathered in other trials may be included in the optimization for robustness. However, optimizations accounting for uncertainty increase the optimization times over simpler optimization criteria substantially. In this work an evaluation with an ED-optimal criterion (accounting for 10% uncertainty in the fixed effect parameters) took ~ 21 times longer than the same with a D-optimal criterion (results not shown). An approach to make ED-optimal design feasible would be to first identify a trial design among several candidate designs to optimize that design using an ED-optimal criterion. Model-based adaptive OD could also be used, where an initial smaller cohort is optimized based on prior information and subsequent cohorts use updated information gathered during previous cohort optimizations [38].

## Conclusion

In conclusion, competing reduced study designs for a TMDD model were optimized using OD methodology and the performance of the designs was assessed and compared to a reference design using several performance metrics. The study reveals factors of importance for an adequate design and illustrates the importance of a balanced evaluation using alternative metrics, depending on the purpose of the trial.

## Electronic supplementary material

Below is the link to the electronic supplementary material.

Supplementary material 1 (DOCX 23 kb)
