# Supplementary material for: Reduced and optimized trial designs for drugs described by a target mediated drug disposition model
Source: J Pharmacokinet Pharmacodyn. 2018 Jun 8;45(4):637–47. doi: 10.1007/s10928-018-9594-9 (PMC6061097; doi:10.1007/s10928-018-9594-9)
Supplement: Supplementary file 1 — Supplementary material 1 (DOCX 23 kb) [file 10928_2018_9594_MOESM1_ESM.docx]

The model used in *Reduced and Optimized Trial Designs for drugs described by a Target Mediated Drug Disposition Model*  by Brekkan et al. can be described as follows:

$$\frac{d{SC}_{D}}{dt}= {-k}_{a}*{SC}_{D}$$

$$\frac{d{OMA}_{T}}{dt}= k_{a}*{SC}_{D}-{CL}_{OMA}*C_{OMA,F}-{CL}_{COMP}*C_{COMP}$$

$$\frac{d{IGE}_{T}}{dt}= k_{syn}-{CL}_{IGE}*C_{IGE,F}-{CL}_{COMP}*C_{COMP}$$

$$K_{d}=K_{d0}*\left( \frac{{OMA}_{T}}{{IGE}_{T}} \right)^{\alpha}$$

$$COMP= 0.5*\left( \left( \frac{K_{d}V_{OMA}V_{IGE}}{V_{COMP}}+{OMA}_{T}+{IGE}_{T} \right)-\sqrt{\left( \frac{K_{d}V_{OMA}V_{IGE}}{V_{COMP}}+{OMA}_{T}+{IGE}_{T} \right)^{2}-4{OMA}_{T}{IGE}_{T}} \right)$$

$$C_{COMP}= COMP/V_{COMP}$$

$$C_{OMA, F}= \frac{{OMA}_{T}-COMP}{V_{OMA}}$$

$$C_{IGE, F}= \frac{{IGE}_{T}-COMP}{V_{IGE}}$$

$$C_{OMA, T}= C_{OMA, F}+C_{COMP}$$

$$C_{IGE, T}= C_{IGE, F}+C_{COMP}$$

where SC_D_ is the amount of drug in the subcutaneous (SC) dosing compartment, OMA_T_ is the nanomolar amount of total OMA, IGE_T_ is the nanomolar amount of total IgE, K_d_ is the dissociation constant, K_d0_ is the dissociation constant when the ratio of OMA_T_ and IGE_T_ equals 1, α is an estimated exponent that accounts for different complexes being formed at different concentrations of IgE and OMA^1^, COMP is the amount of complex, C_COMP_ is the concentration of complex, C_OMA,F_ is the concentration of free OMA, C_IGE,F_ is the concentration of free IgE (IGE_F_), C_OMA,T_ is the concentration of OMA_T_, C_IGE,T_ is the concentration of IGE_T_, k_a_ is the absorption rate constant, CL denotes non-target related clearance and V denotes volumes. C_IGE,F_, C_IGE,T_ and C_OMA,T_ were measured in the study. Inter-individual variability (IIV) of model parameters is described according to log-normal distributions and residual variability with an additive error component in the log domain. Parameter values for the model can be seen in Table 1A below.

Table 1A: Parameter estimates of the model used for evaluation and optimizations.

| Parameter (unit) | **Description** | **Estimate** |  |
| --- | --- | --- | --- |
| **Structural model**  CL_OMA_ (mL h^-1^) | OMA clearance | 7.32 |  |
| V_OMA_ (mL) | OMA central volume | 5900 |  |
| V_IGE_ (mL) | IgE central volume | 5900 |  |
| k_a_ (h^-1^) | Absorption rate constant | 0.02 |  |
| CL_IGE_ (mL h^-1^) | IgE clearance | 71 |  |
| k_syn_ (nm h^-1^ ) | IgE synthesis rate | 0.158 |  |
| CL_Comp_ (mL h^-1^) | Complex clearance | 5.86 |  |
| V_COMP_ (mL) (U mL^-1^) | Complex central volume | 3630 |  |
| K_d0_ (nm mL^-1^) | Equilibrium dissociation rate | 0.00107 |  |
| α^a^ (unitless) | Scaling factor for K_d_ | 0.157 |  |
| **Inter-individual variability (IIV)^b^**  ω_CLOMA_ |  | 0.20 |  |
| ω_VOMA_ |  | 0.13 |  |
| ω_Ka_ |  | 0.40 |  |
| ω_CLIGE_ |  | 0.25 |  |
| ω_ksyn_ |  | 0.23 |  |
| ω_CLcomp_ |  | 0.35 |  |
| ω_Vcomp_ |  | 0.25 |  |
| ω_Kd0_ |  | 0^*^ |  |
| **Residual variability^c^**  σ_OMAT_ |  | 0.17 |  |
| σ_IGET_ |  | 0.21 |  |
| σ_IGEF_ |  | 0.22 |  |
| OMA = Omalizumab, IgE = Immunoglobulin E, OMA_T_ = Total OMA concentration,  IGE_T_ = Total IgE concentration,IGE_F_ = Free IgE concentration.  ^a^ α scales K_d_ by the ratio of OMA_T_ and IGE_T_. When the ratio is 1, K_d_ = K_d0._  ^b^ Interindividual variability reported as coefficient of variation.  ^c^ Residual error reported as coefficient of variation | | | |

1. Liu J, Lester P, Builder S, Shire SJ. Characterization of complex formation by humanized anti-IgE monoclonal antibody and monoclonal human IgE. *Biochemistry* 1995, **34**(33)**:** 10474-10482.
